# The appeal of the PDC program

**DOI:** 10.3389/fpsyg.2013.00236

**Published:** 2013-04-26

**Authors:** Thomas Wasow

**Affiliations:** Department of Linguistics, Stanford UniversityStanford, CA, USA

In 2006, I gave a series of lectures in Paris arguing for a set of rather general strategies governing language production. I was inspired to do this largely by the work of John A. Hawkins (especially his 2004 book), which argues that many morphosyntactic properties of languages can be explained in terms of how they facilitate processing. Hawkins's thesis is that language users favor structures that are easy to parse; that many languages grammaticalize these parsing preferences over time; and that this can explain facts about linguistic typology and language change. This idea is deeply insightful and has been influential among typologists, syntacticians, and psycholinguists. But I have always felt uncomfortable about a number of the particular processing strategies Hawkins proposed, because they struck me as too focused on the problem of parsing—that is, on comprehension. The form that an utterance takes is determined by the speaker, not the listener. A theory that relies too heavily on comprehension-based considerations to explain properties of languages must assume that speakers design their utterances primarily to accommodate their audience's needs, rather than their own. Given the inherent difficulty of the task of articulating thoughts as fast as people do in ordinary speech, more production-oriented explanations in the Hawkinsian style would seem more convincing.

I proposed four general production strategies:

- Contiguity: Avoid interrupting semantic or syntactic units.
- Procrastination: Put off what is hard.
- Brevity: Keep what is predictable short.
- Audience design: Let your audience know when you anticipate difficulty.

I also argued that ambiguity avoidance plays a much smaller role in shaping language structure than is generally assumed. None of these proposals was original with me, though my formulations were somewhat different from any in the literature. I did not try to ground them on any more general facts about human cognition, though they all seemed rather intuitive to me.

I used these four strategies to account for a range of phenomena I had studied, mostly in English, as well as some other results in the literature. For example, Contiguity helps to explain why verb-particle combinations that are semantically opaque (like *figure out*) appear adjacent to one another at significantly higher rates than those that are semantically transparent (like *lift up*); see Lohse et al. (2004). And Brevity helps to account for the fact that, where the subordinator *that* is optional at the start of a relative clause, its occurrence is negatively correlated with the likelihood of having a relative clause in that position; see Wasow et al. (2011). I never tried to publish these lectures, both because they did not present any new data and because I felt others were developing potentially deeper explanations of some of the phenomena I considered.

MacDonald's Production-Distribution-Comprehension project (PDC) is similar in some ways to what I was trying to do in my lectures, but it is more ambitious, for several reasons. First, she is trying to explain facts about language in terms of more general facts about memory and cognition. Second, she takes a stronger stand than I did, explicitly saying that production demands are the driving force behind language structure and change. These facts make PDC an exciting research program, and she does a good job of arguing for its plausibility. It also converges nicely with some developments in theoretical linguistics. In particular, her conception of “sentences, phrases, and words as plans and sub-plans” (MacDonald, 2013) comports well with recent work in construction grammar (see e.g., Goldberg, 1995, 2006; Boas and Sag, 2012).

There are obvious points of similarity between my proposed production strategies and the production biases MacDonald discusses. Most obviously, her Easy First and my Procrastination say the same thing. In addition, if what I call semantic and syntactic units are plans, in her sense, and interrupting a plan is a form of interference, then Contiguity would follow from Plan Reuse and Reduce Interference. Admittedly, this requires a broader construal of these biases than MacDonald presents in her paper, but it seems like a natural extension. My Brevity strategy doesn't seem to follow from MacDonald's proposals, but it does seem like something that could reduce the memory demands in production. We agree that audience design plays a role in production (MacDonald, 2013), but that its role is much smaller than many other researchers have suggested; note in particular how limited my strategy is. Finally, we also agree that ambiguity avoidance is overrated.

Despite my enthusiasm for the PDC program, it faces many challenges. In my remaining space, I will sketch three.

The first is one that MacDonald herself raises, namely, the fact that certain languages (notably Japanese and Korean) exhibit a long-before-short preference—just the opposite of what Easy First predicts. MacDonald (2013) suggests that this can be accounted for in terms of Plan Reuse because, “tendencies for ordering object and recipient noun phrases reflect the adaptation of plans from more common sentences with only one noun phrase.” But this can't account for the different behaviors of SVO and SOV languages with respect to the ordering of long, complex constituents, for “sentences with only one noun phrase” have the same structure in both languages, namely, SV.

Intuitively, what drives both the short-before-long tendency in languages like English and the long-before-short tendency in languages like Japanese is the extra difficulty caused by dealing with complex tasks in the middle of some other task. This poses problems for both production and comprehension, and is exemplified by the awkwardness of sentences like (1a) in comparison to (1b) and (1c):

1a. Is that the climate is changing an established fact?
1b. That the climate is changing is an established fact.
1c. Is it an established fact that the climate is changing?

Having the subordinate clause *that the climate is changing* either at the beginning or the end of the main clause makes the sentence substantially more natural. Arguably, this could be subsumed under Contiguity, if the main clause is regarded as a syntactic and semantic unit. (1b) is also somewhat more awkward than (1c), and this can be attributed to Easy First (or, equivalently, Procrastination). In strictly verb-final languages like Japanese, however, putting long, complex constituents at the end of their clause is simply ungrammatical. Hence, the choice is between having such constituents at the beginnings of clauses or in the middle. The beginning is preferable (even though it violates Easy First) because it avoids the processing of the complex constituent in the middle of a clause (that is, it conforms to Contiguity). Note that, in verb-medial languages, the ordering preferences of Easy First and Contiguity can be accommodated simultaneously, by putting complex constituents at the ends of clauses; but in strictly verb-final languages, clause-final position is not available, so only Contiguity can be accommodated, not Easy First. It is interesting in this regard that Hawkins (1994; p.144) notes that the long-before-short tendency for Japanese is weaker than the short-before-long tendency in languages like English.

A very different kind of challenge to MacDonald's position is the observation that one kind of ambiguity is systematically avoided in languages, namely, ambiguity with respect to basic argument structure—that is, who did what to whom. In the colorful words of Fries (1940), “If, for example, we are to say anything about a bear and a man in connection with the action of killing, it is ‘essential and unavoidable’ that we indicate which one did the killing and which one was killed.”^1^ It is striking that natural languages consistently have grammatical mechanisms to mark argument structure, and that instances of ambiguity with respect to argument structure are relatively rare. Clearly, avoidance of this sort of ambiguity facilitates comprehension, but it is far from obvious how to give it a production-based explanation. In formulating an utterance, the speaker knows whether Fries's man or his bear is the killer. Yet languages consistently mark this distinction, presumably because it is so important for communication. Nothing in PDC makes it obvious why this sort of ambiguity should be avoided.

My final challenge for the PDC is based on Futrell's (2010) insightful account of the function of grammatical noun classes. Noun classes are sets of nouns that have morphosyntactic properties in common (for example, particular agreement or case-marking patterns) but do not form a semantically coherent class. Familiar instances are the grammatical gender systems of many European languages (including French, German, Spanish, and Russian). Such systems abound across a wide variety of language families. At first glance, noun classes seem to serve no function, as evidenced by the fact that many languages (including English) get along without them. Moreover, they clearly add complexity to the task of language learning—as any adult English speaker who has studied a language with noun classes knows. The existence of so many languages with noun classes is therefore a puzzling fact, crying out for explanation.

Futrell makes a persuasive case that the marking of noun classes on preceding words (notably agreement on determiners and adjectives) makes nouns more predictable. For example, in a language with three genders, the occurrence of a feminine article reduces the possible following nouns to just those that are grammatically feminine. This clearly can facilitate comprehension by providing early information about upcoming material, thereby reducing the search space during the identification of nouns. It may have some benefit for the speaker—say, in lexical retrieval—but it also puts an extra burden of advance planning on the speaker, who must select the gender of the upcoming noun before uttering the preceding article. Thus, it seems to me that a functional explanation of the widespread existence of languages with noun classes is much more convincing if it is based on comprehension, not production. In particular, explaining the abundance of languages with noun classes is a challenge for PDC.

Let me emphasize that these three examples are intended only to raise questions. I do not wish to suggest that they are definitive counterexamples to PDC. Rather, they are cases in which I believe that comprehension-based explanations of cross-language generalizations seem more natural than production-based explanations. I hope that they can be accommodated within PDC or some similar theory. Insofar as properties of languages can be accounted for on the basis of general properties of human cognition, memory, and efficient communication, genuine explanation will be achieved. PDC is a step in that direction.

## Footnotes

^1^Fries put “essential and unavoidable” in quotes because he borrowed the phrase from Sapir (1921).
